# Novel Flp pilus biogenesis-dependent natural transformation

**DOI:** 10.3389/fmicb.2015.00084

**Published:** 2015-02-10

**Authors:** Angel Angelov, Paul Bergen, Florian Nadler, Philipp Hornburg, Antoni Lichev, Maria Übelacker, Fiona Pachl, Bernhard Kuster, Wolfgang Liebl

**Affiliations:** ^1^Lehrstuhl für Mikrobiologie, Technische Universität MünchenFreising-Weihenstephan, Germany; ^2^Lehrstuhl für Proteomik und Bioanalytik, Technische Universität MünchenFreising-Weihenstephan, Germany

**Keywords:** natural transformation, tight adherence pili, *Micrococcus luteus*

## Abstract

Natural transformation has been described in bacterial species spread through nearly all major taxonomic groups. However, the current understanding of the structural components and the regulation of competence development is derived from only a few model organisms. Although natural transformation was discovered in members of the *Actinobacteria* (high GC Gram-positive bacteria) more than four decades ago, the structural components or the regulation of the competence system have not been studied in any representative of the entire phylum. In this report we identify a new role for a distinct type of pilus biogenesis genes (*tad* genes, for *t*ight *ad*herence), which so far have been connected only with biofilm formation, adherence and virulence traits. The *tad*-like genes found in the genome of *Micrococcus luteus* were shown to be required for genetic transformation in this actinobacterial species. We generated and analyzed individual knockout mutants for every open reading frame of the two predicted *tad* gene clusters as well as for a potential prepilin processing peptidase and identified the major component of the putative pili. By expressing a tagged variant of the major prepilin subunit and immunofluorescence microscopy we visualized filamentous structures extending from the cell surface. Our data indicate that the two *tad* gene islands complementarily contribute to the formation of a functional competence pilus in this organism. It seems likely that the involvement of *tad* genes in natural transformation is not unique only for *M. luteus* but may also prove to be the case in other representatives of the *Actinobacteria*, which contains important medically and biotechnologically relevant species.

## Importance

In almost all naturally transformable bacteria, proteins building special transport systems (type II secretion) and cell appendages (type IV pili or pseudopili) have been implicated in the uptake of external DNA into the cell. This study shows that a special type of pilus biogenesis genes (*tad* genes), which so far have only been connected with adherence and virulence but never before with genetic transformation, are required for transformation in *Micrococcus luteus*. Also, this is the first report about the transformation machinery of a representative of the phylum of high-GC Gram-positive bacteria (*Actinobacteria*). This has implications for the discovery of new transformable species in this phylum lineage, among them some important pathogens.

## Introduction

Genetic transformation is one of the ways for the entry of exogenous genetic material in bacterial cells and one of the main routes for horizontal gene transfer—a phenomenon which is involved in the adaptation to new environments, the spread of antibiotic resistance traits and which is thought to promote rapid evolution. The ability to undergo transformation has been observed in bacterial species belonging to several major taxonomic groups, and some of them have been established as models to study this complex process. The regulation of natural competence development and the structural components of the DNA uptake machinery are best characterized in *Neisseria gonorrhoeae*, *Haemophilus influenzae*, *Vibrio cholerae*, *Thermus thermophilus*, *Bacillus subtilis*, and *Streptococcus pneumoniae* (Chen and Dubnau, 2004; Hamilton and Dillard, 2006; Maughan et al., 2008; Averhoff, 2009; Seitz and Blokesch, 2013). Historically, *M. luteus* (“*M. lysodeikticus*”) strains have been among the first bacteria used to study natural transformation (Kloos, 1969a,b; Kloos and Schultes, 1969), but interestingly this seems to have been “forgotten” in the scientific literature of the last decades where the species is not listed in comprehensive reviews about the topic. The competence system of neither *M. luteus* nor of any other bacterium belonging to the *Actinobacteria* phylum (high-GC Gram-positive bacteria), which contains a number of important pathogens, has been studied in detail.

Despite many differences in detail, common features of all competence systems studied so far are (i) the requirement for proteins with similarity to type IV pilus (T4P) biogenesis or type II or rarely type IV secretion system (T2SS, T4SS) components and (ii) the presence of a DNA translocation complex at the cytoplasmic membrane. In this context it is interesting to note that some phylogenetic relatedness appears to exist between T2SS assembling T4P and T4SS assembling conjugative pili (Chagnot et al., 2013). In those naturally transformable bacteria that also have T4P, correlation has always been shown between the presence of pili and competence. *S. pneumoniae* produces long (2–3 μm) pili which bind DNA (Laurenceau et al., 2013), but for most transformable species there is a lack of evidence for a direct role of the pili (such as interaction with DNA) in transformation. The requirements for transformation of T4P or T2SS genes also in organisms that lack obvious pilus structures (such as *B. subtilis*) has led to the idea of the “competence pseudopilus”—a structure similar to but still distinct from T4P, whose function is to enable the transforming DNA to gain access to the translocation complex at the cytoplasmic membrane (Chen and Dubnau, 2003, 2004).

Flp (fimbrial low-molecular weight protein) pili constitute a distinct class of cell appendages in bacteria. Their major prepilin subunits form a monophyletic group and a unique subclass within the type IVb prepilin family (Kachlany et al., 2001; see Imam et al., 2011). The features that distinguish Flp prepilin subunits from the subunits of other pili are their small size (a length of 50–80 amino acids, as opposed to approximately 200 amino acids for the Pil or Com prepilins) and the presence of an N-terminal conserved “Flp-motif” which is found at the N-terminal side of a hydrophobic stretch of about 20 amino acids. Flp prepilins are cleaved by a dedicated prepilin peptidase, TadV, after the glycine residue of a conserved processing site (G/XXXXEY), and this processing is essential for pilus assembly and function (Tomich et al., 2006). TadV is distinguished from other T4P prepilin peptidases by the lack of an N-terminal methyltransferase domain. Almost always, Flp prepilin-encoding genes are found within *tad* (*t*ight *ad*herence) loci, together with pilus biogenesis genes. Despite the relatively broad distribution of *tad* gene clusters in bacteria (Imam et al., 2011), published experimental evidence for the presence of Flp pili is limited to *Aggregatibacter (“Actinobacillus”) actinomycetemcomitans* (Rosan et al., 1988; Kachlany et al., 2001), *Caulobacter crescentus* (Skerker and Shapiro, 2000) and *Pseudomonas aeruginosa* (de Bentzmann et al., 2006)*. tad* genes have been shown to be essential for adherence-associated traits like biofilm formation, colonization and virulence (Tomich et al., 2007). To our knowledge, other functions have not been reported so far.

We noticed that the *M. luteus* genome contains *comEC* and *comEA* orthologs, but lacks genes with similarity to known competence genes from other naturally transformable bacteria. Therefore, we decided to initiate a study on competence development in this bacterium. In this report, we show that Flp pili are expressed in *M. luteus* and identify Flp piliation-related *tad* genes organized in two distinct loci on the chromosome of *M. luteus* as absolutely required for natural transformation and pilus formation.

## Results

### Competence development in *M. luteus*

**Figure 1:**
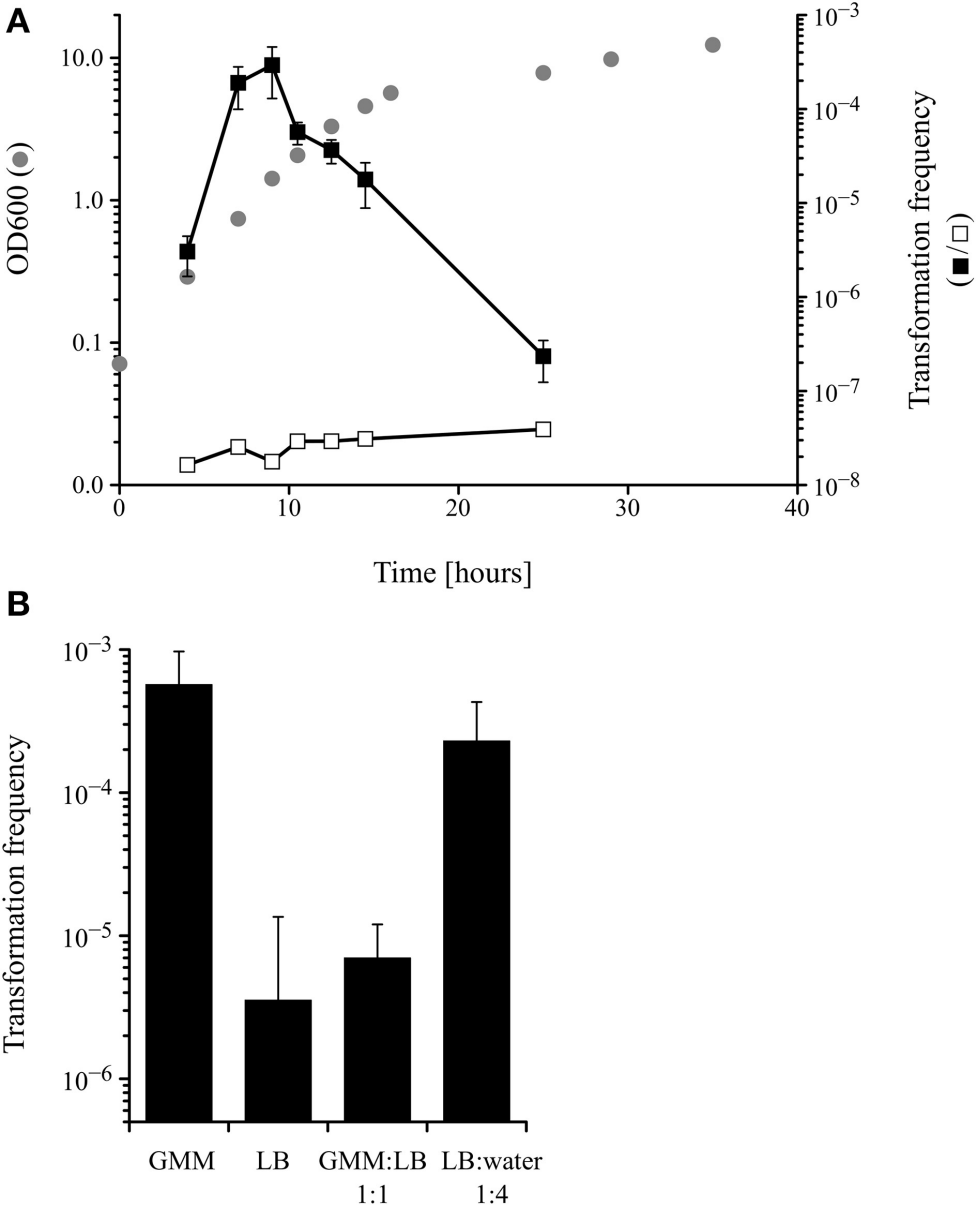
**(A)** Kinetics of competence development during growth of *M. luteus* trpE16 in glutamate minimal medium (GMM) at 30°C. Optical density (gray circles), transformation frequency (black squares) and the frequency of spontaneous reversion of the trpE16 strain to Trp^+^ (white squares) were measured at the indicated time points. **(B)** Effect of growth medium on the transformation frequency of *M. luteus* trpE16. The cells of one culture grown in complex medium were split in the respective media at cell density of 10^8^ × ml^−1^ and incubated for 16 h before determining the transformation frequency. The mean values and standard deviation of three (in **A**) and four (in **B**) independent experiments are shown. GMM, glutamate minimal medium; LB, lysogeny broth medium.

Competence for DNA uptake from the surrounding media is often an inducible and transient physiological state that is subject to complex regulation. Because the only data about competence development in *M. luteus* date back to the late 1960s (Kloos, 1969b; Kloos and Schultes, 1969), it was important to analyse the transformability of the *M. luteus* strain used in this work during growth in a defined minimal medium. The transformation frequency, measured by transforming the tryptophan auxotrophic strain trpE16 to prototrophy, peaked during mid to late exponential growth phase, with a maximum at around OD 1 (Figure 1A). Additional experiments showed that competence in *M. luteus* was also affected by the nutritional state, since cells grown in complex media (LB) displayed approximately 100-fold lower transformation frequencies than those grown in minimal medium (GMM), similar to previous results reported by Kloos (1969b). Growth in diluted complex media led to restoration of the high transformation rates observed in minimal medium-grown cells (Figure 1B).

These experiments indicate that competence development in *M. luteus* is a regulated trait reminiscent of the situation described for other competent bacteria, e.g., *B. subtilis* and *S. pneumoniae*. However, because no detectable homologs to proteins of the *B. subtilis* quorum sensing regulatory cascade could be found in the genome of *M. luteus* (apart from ComA; no homologs to ComX, ComP, ComS, ComQ, ComK or PhrC), the molecular details of competence regulation must be different and remain to be unraveled in *M. luteus*.

### Identification of putative pilus biogenesis gene clusters in the genome of *M. luteus*

A systematic search of the genome of *M. luteus* for ORFs with sequence similarity to known competence genes/gene products from prototypical naturally transformable species led to the identification of putative orthologs of the *B. subtilis* ComEA, ComEC, and DprA proteins (Mlut_12460, Mlut_12450, and Mlut_09260), but failed to uncover further candidate competence genes. Expectedly, deletion of the *M. luteus* ComEC-encoding sequence led to the complete loss of transformability, as we have shown previously (Angelov et al., 2013). Because a link between transformation and macromolecular transport machineries (usually T4P or T2SS proteins) has been demonstrated for most of the model systems, we searched for possible pilus biogenesis gene clusters in *M. luteus*. Also, we searched for ORFs whose putative products display characteristics typical for pilins using the *pilfind* program (Imam et al., 2011) as well as by manual inspection of the predicted proteome. Two separate gene clusters were found in the genome of *M. luteus*, termed *tad-1* (Mlut_07500 to Mlut_07560) and *tad-2* (Mlut_18150 to Mlut_18190), both of which contain ORFs with sequence similarity to the prototypical Flp pili biogenesis proteins, Flp pilin and Flp-like proteins of *A. actinomycetemcomitans* (BLAST *E*-value cutoff of 10^−5^; Kachlany et al., 2000). Analysis of these clusters showed that in both of them the predicted genes were organized in the order *tadA*-*tadB*-*tadC*-(*flp*)-*tadE/F*-*tadG* (*A. actinomycetemcomitans* nomenclature). In the *tad-2* cluster the TadB- and TadC-encoding ORFs were fused into one ORF. The *M. luteus tad-2* gene cluster contained two ORFs with the conserved Flp processing site (G/XXXXEY) but only one of these, Mlut_18170, was predicted to encode a protein with a Flp prepilin-typical size (77 amino acids) and we therefore named the corresponding gene *flp*. The ORFs with Flp pilin-like features encoded in the *tad-1* cluster displayed a deviating N-terminal processing site (G/XXXXE[FS]) and a size of about 150 amino acids (Table 1), thus showing characteristics of TadE/F minor pilins.

**Table 1:**
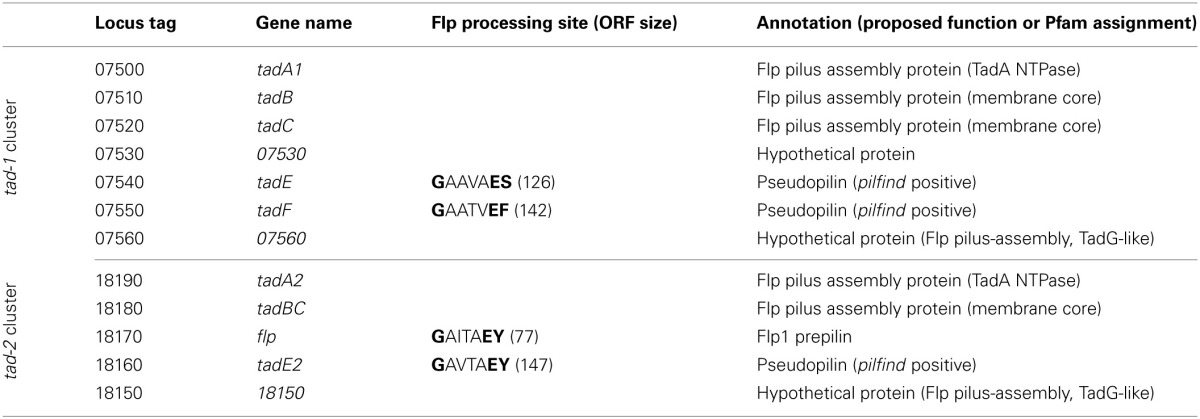
Genes of the *tad* gene clusters of *M. luteus* trpE16 identified as required for natural transformation^*^.

**Figure 2:**
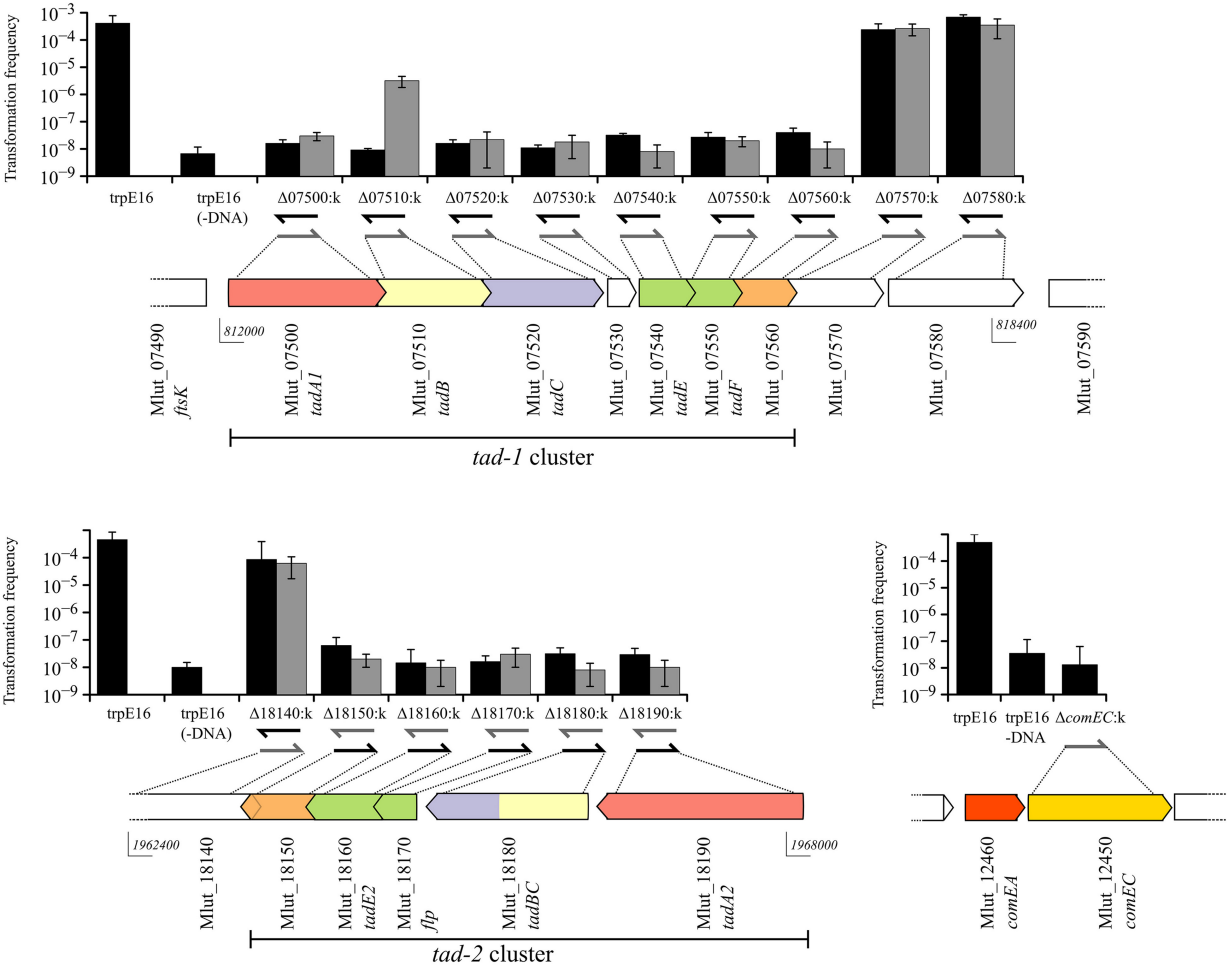
**Genetic organization of the two *tad* clusters and of the *comEA/EC* locus of *M. luteus* and transformation frequencies of the respective gene deletion mutants (gray bars for kanamycin gene insertion in the same orientation and black bars for insertion opposite to the respective ORF)**. The *in silico* predicted Flp and Flp-like genes are shown in green and the pili biogenesis genes in red, yellow and blue for *tadA*, *B*, and *C*, respectively. Arrow-arrow contacts indicate potential translational coupling of the ORFs (sequence overlap). The orientation of the kanamycin resistance gene in the mutants is shown by arrows below the bars. The bars are mean values for four independent transformation assays and the error bars are standard deviation (SD).

* tadB is not essential for transformation, but its deletion led to a >100-fold decrease in transformation frequency (see Figure 2).

A putative prepilin peptidase gene (*tadV*) was missing in both *M. luteus tad* loci, but this seems to be typical for the organization of the *tad* genes also in other actinobacteria (Tomich et al., 2007). We found only one ORF in the genome of *M. luteus*, Mlut_05160, which codes for a protein with characteristics typical for a Flp prepilin peptidase, i.e., with two highly conserved aspartate residues, five predicted transmembrane domains and the lack of an N-terminal methyltransferase domain.

### Introduction of mutations into the *M. luteus tad* gene clusters and evaluation of the resulting phenotypes

In order to assess their putative role in transformation, we generated deletion mutants for each of the ORFs in the two *M. luteus* ATCC 27141-trpE16 *tad* gene clusters and for the putative Flp prepilin peptidase ORF by individually replacing most of their coding sequences with a kanamycin resistance marker. In addition, ORFs flanking the predicted *tad* cluster genes were deleted in the same manner. All mutations were introduced in the tryptophan auxotrophic strain trpE16. For each of the ORFs of the two *tad* loci, two sets of mutants were generated: one set with the kanamycin gene in the same orientation as the disrupted ORF and the other set with the resistance marker in the opposite orientation.

The frequencies of transformation to prototrophy were measured for all strain trpE16-derived gene replacement mutants, using donor DNA from the Trp^+^ wild type strain ATCC 27141. As can be seen from Figure 2, deletion of any of the ORFs, with the exception of Mlut_07510, in any of the predicted *tad* clusters led to the loss of transformability irrespective of the orientation of the kanamycin marker. For the ORF Mlut_07510, a >100-fold reduction in transformation frequency was observed when the antibiotic resistance marker was inserted in the same orientation as the ORF while the cells were non-transformable when the kanamycin gene was in the opposite orientation relative to the disrupted ORF. Considering that replacement of Mlut_07510 by the kanamycin cassette in one of the orientations reduced but not completely abolished transformability, we conclude that this ORF is not essential for transformation.

Overall, these results showed that gene products from both *tad* clusters are required for competence in *M. luteus*. Because polar effects of the kanamycin cassette insertion on downstream genes cannot be excluded, it is currently not clear if each gene of both clusters is required for competence. However, considering that mutations of the most distal genes of the *tad* clusters led to loss of transformability, it is clear that both clusters are required for competence in *M. luteus*.

### Visualization of competence-related pili

In wild type *M. luteus* cells, initially we could not visualize any pilus structures by electron microscopy and could not detect putative pilin proteins in sheared extracellular fractions of competent cultures using SDS-PAGE (type IV pili and other extracellular appendages can often be detached from the surface of bacteria by vortexing). This could be due to a limited expression of pili only in a fraction of the cells in a population, or to an undetectable protrusion of the competence (pseudo)pili beyond the cell surface. For a more sensitive pili detection, we introduced a gene for a C-terminally tagged version of the predicted major Flp pilin subunit into the *M. luteus* genome. This was achieved by replacing the wild type *flp* gene (Mlut_18170) with a *flp-Strep*-tag II fusion allele. The modifications in this strain left the genomic region upstream of *flp* unchanged to ensure transcription of the Flp-Strep-encoding gene fusion by the native *flp* promoter, while the ORF downstream of *flp* (Mlut_18160) was placed under the control of the promoter of the kanamycin cassette. The newly constructed strain (Flp-Strep:k) was transformable, albeit at a fivefold lower frequency than the parental strain (Table 2), confirming that the modified pilin had retained its function with respect to competence development.

**Table 2:** **Transformation frequency of the *M. luteus* strain Flp-Strep:k**.

**Sample**	**Transformation frequency (±SD)**
trpE16	4.5 ± 8 × 10^−4^
Flp-Strep:k	1.0 ± 6 × 10^−4^
trpE16 (-DNA)	<10^−8^

*The assays were performed with four independent cultures for each sample (n = 4)*.

**Figure 3:**
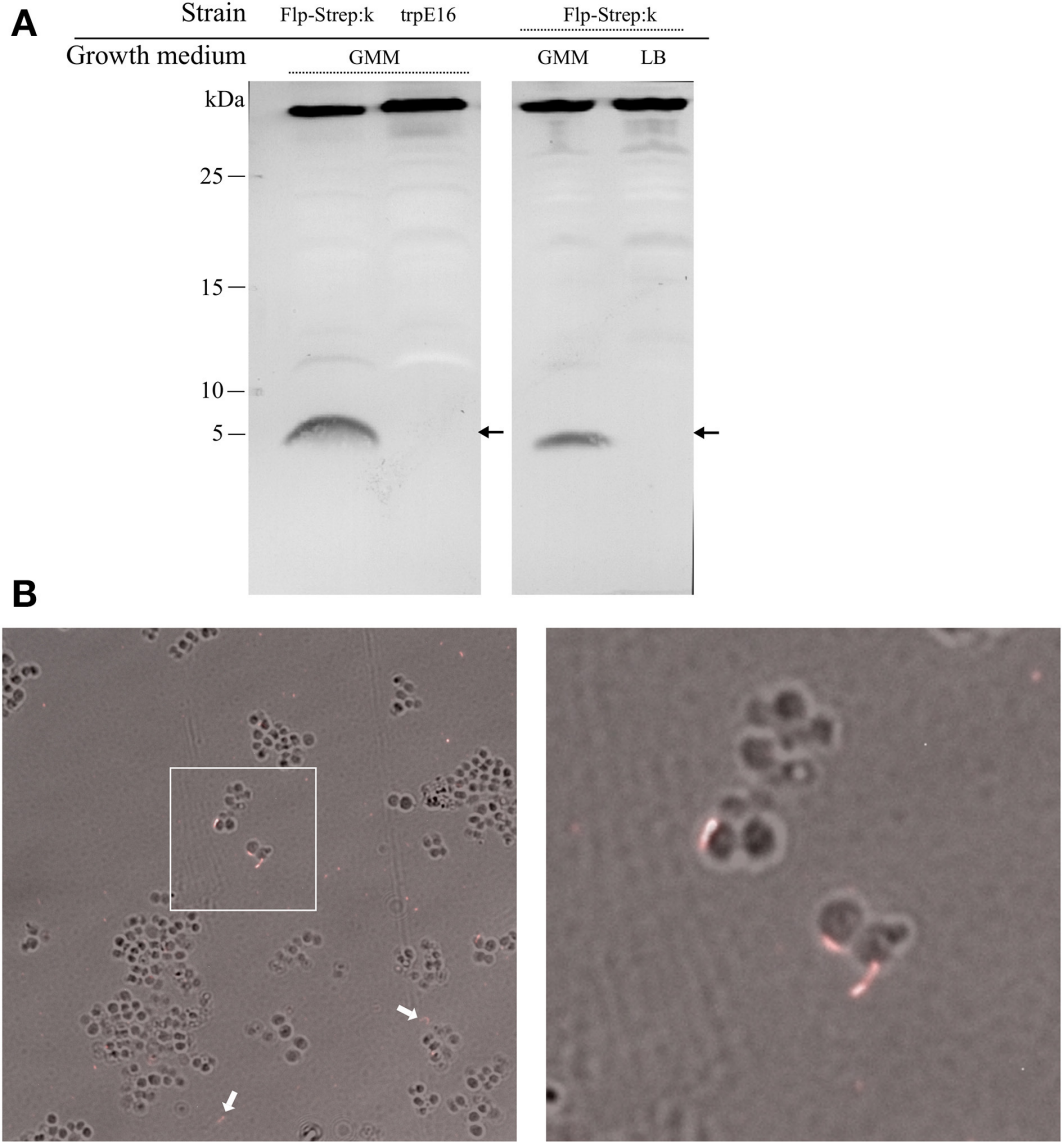
**Western blot analysis and visualization of competence-related pili in *M. luteus* by immunofluorescence microscopy**. **(A)** Alkaline phosphatase-conjugated *Strep*-Tactin Western blot of extracellular sheared fractions of the strain carrying an affinity-tagged version of the Flp prepilin (Flp-Strep:k) and the wild type srtain (trpE16), grown under competence-inducing conditions (left panel). Extracellular sheared fractions of the Flp-Strep:k strain grown in minimal (GMM) and in rich (LB) medium, showing production of Flp-Strep pilin only under competence-inducing conditions (right panel). The predicted size of the Flp-Strep fusion is 5.6 kDa (arrows). The band around 35 kDa, originating from an unknown biotinilated *M. luteus* protein, was included to demonstrate equal amount of proteins in the samples. **(B)** Immunofluorescence microscopy of the Flp-Strep:k strain, grown to competence state, using an Oyster 556-labeled anti-*Strep*-tag antibody. Shown are overlays of fluorescence and phase contrast images. The white arrows show cell-unassociated pili. The right panel is a magnification of the white square area of the left panel.

We could detect processed Flp-Strep pilin subunits (predicted mass of 5.6 kDa) by Western blot analysis of the extracellular sheared fraction of the Flp-Strep:k strain. Also, Flp-Strep pilin was expressed under competence-inducing conditions, e.g., when the cells were grown in minimal medium, while no protein could be detected in rich medium (Figure 3A).

Supporting the results from the Western blotting experiments, immunofluorescence microscopy of Flp-Strep:k cells grown under competence-inducing conditions with fluorescently labeled anti-*Strep*-tag II antibodies revealed the presence of short filamentous structures (Figure 3B). On average, fluorescent Flp structures were found on 3% of the Flp-Strep:k cells when the culture was grown under conditions optimal for competence (4307 cells counted). Often, also fluorescent appendages not associated with cells were observed, which may result from their shearing off during the pipetting and centrifugation steps. No fluorescent foci were observed in cells grown in rich medium or to the stationary phase (non-competence conditions). Also, microscopy with trpE16 as a control strain showed no fluorescent appendages (approximately 5000 cells screened). Taken together, the data from the Western blot and immunofluorescence microscopy experiments showed the existence of an extracellular Flp structure in *M. luteus* and suggested that Flp expression is also a transient trait, co-occurring with competence. In *S. pneumoniae*, the single so far reported case of direct visualization of a transformation pilus in Gram-positive bacteria, almost all cells appear to harbor one or a few ComGC appendages on the cell surface (Laurenceau et al., 2013). In contrast, only a small subpopulation of the *M. luteus* cells seemed to bear Flp appendages under competence-inducing conditions.

### Surface exposition and identification of the major pilin subunit

**Figure 4:**
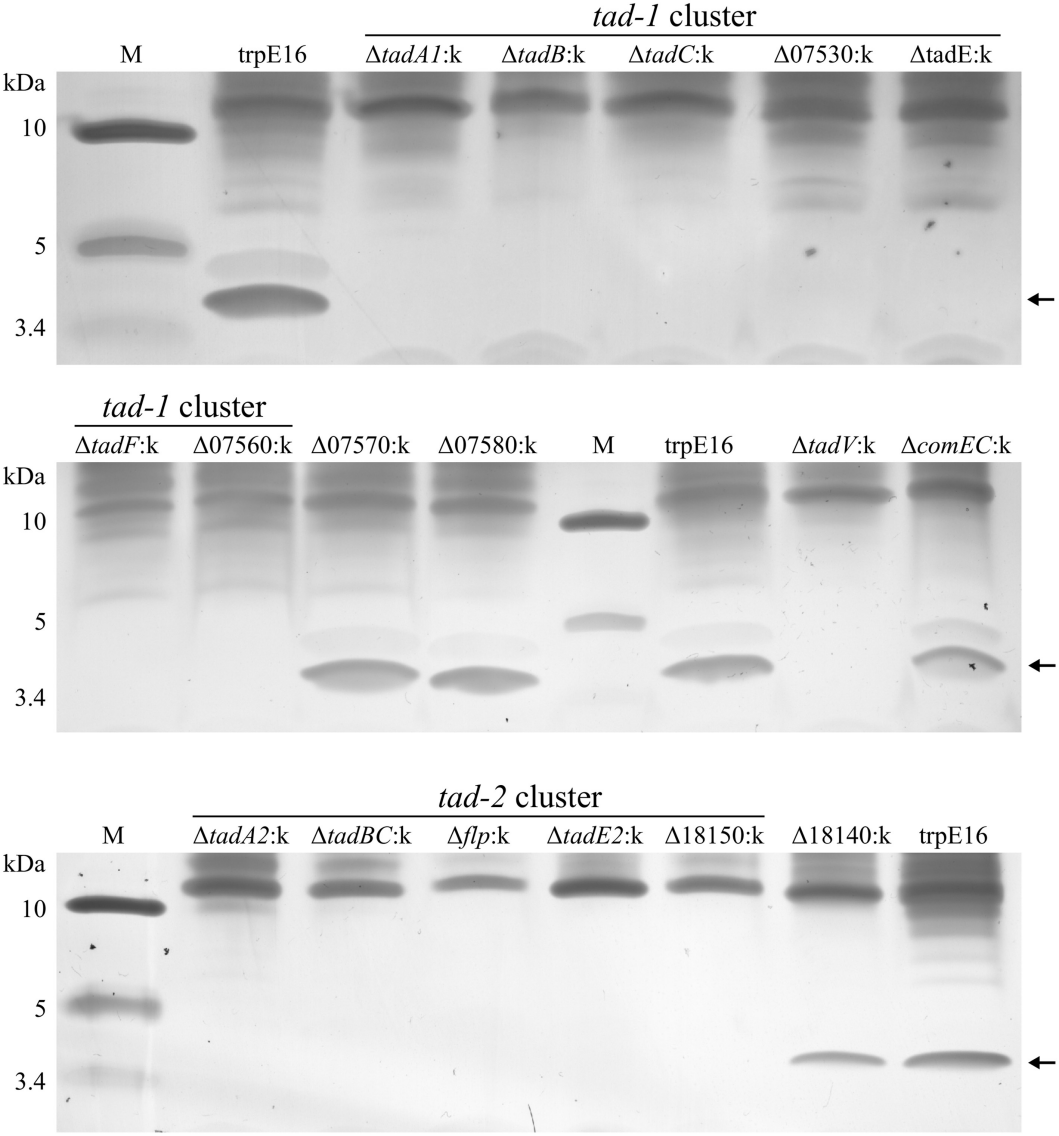
**Tris-tricine SDS-PAGE analysis (silver staining) of extracellular sheared fractions from *M. luteus* trpE16 and selected non-polar *tad* gene disruption mutants**. All the strains were grown under competence-inducing conditions and the preparation of enriched high molecular weight protein complexes from extracellular fractions was performed in an identical manner. The amount of sample applied per lane was normalized based on total protein content in the preparations.

After we established that Flp appendages are expressed on only a small fraction of the *M. luteus* cells in a competent population, we attempted to concentrate and partially purify these structures from extracellular sheared fractions using ultrafiltration (molecular weight cutoff of 100 kDa). With this approach, after concentrating the extracellular fraction approximately 100-fold and using silver staining, we could detect putative pilin subunits by Tricin-PAGE analysis which had the predicted molecular weight of the processed major pilin Flp (4.4 kDa) and was absent in the respective deletion mutant, Δflp:k (Figure 4). Using in-gel proteolytic digestion and mass spectrometry we could identify Flp protein fragments in excised gel pieces of the 4.4 kDa band, confirming that Flp (encoded by Mlut_18170 in the *tad-2* cluster) is the major constituent of these extracellular high molecular weight structures (Supplementary Table 1). Fragments from other proteins, including apparently intracellular enzymes probably originating from lysed cells, could also be detected in small abundance in our preparations. The predicted minor pilin subunits TadE/E2 and TadF could not be detected in the enriched samples by the LC-MS/MS method used in this study, despite the extremely high sensitivity of the method (Pachl et al., 2013), suggesting that they are not a part of the sheared high-molecular weight complex.

Having confirmed the identity of the 4.4 kDa protein band, we used the above shearing/enrichment method and Tricin-PAGE to examine if Flp subunit polymers were present on the surface of the previously generated *tad* gene cluster mutants. Extracellular Flp complexes could not be detected in the prepilin peptidase (ΔtadV:k) or in any of the deletion mutants in the *tad-2* cluster. Also, all of the *tad-1* cluster mutants were unable to build high molecular weight Flp assemblages on the cell surface, detectable with our shearing/enrichment—silver stain method (Figure 4). In summary, this experiment showed that both *tad* clusters are required for the formation of extracellular Flp pilin complexes in *M. luteus*. In the *tad* deletion mutants examined by us there was a complete correlation between transformability and the presence of Flp pilin complexes, i.e., the lack of high molecular mass Flp structures on the cell surface was always accompanied by a lack of transformability.

## Discussion

This work for the first time shows that Flp pili, which so far have only been associated with adherence, biofilm formation and virulence traits, can play an essential role in natural transformation. We could not observe any obvious differences in adherence, cell aggregation or microcolony morphology between the wild type and any of the *tad* mutant *M. luteus* strains, which may mean that the main physiological role of the Flp pili in this organism is in transformation.

The gene deletion experiments clearly showed that in *M. luteus* the products of two distant *tad* loci, *tad-1* and *tad-2*, which are about 1.2 Mbp apart on the chromosome, are simultaneously needed for competence. Although each of the two *tad* gene clusters apparently contains a complete set of Flp prepilin and pilus biogenesis genes, deletions in any of the *tad* genes, with the exception of *tadB*, led to a complete loss of transformability. Transformation of the *tadB* (Mlut_07510 within the *tad-1* locus) deletion mutant was also largely inactivated, but low-level transformability was still detectable with our assay which may be due to a partial complementation of its function by the paralogous *tadB/C* gene of the *tad-2* cluster. *M. luteus* TadB and TadB/C are probably the membrane components of the Flp pilus, considering their similarity to members of the PilC family of proteins (Peabody et al., 2003) and the presence of five and three transmembrane domains, respectively. Inspection of the primary sequence of the two distal ORFs of the *tad* clusters which we determined to be essential for transformation, Mlut_07560 and Mlut_18150, revealed very weak similarity in their N-terminal regions to members of the TadE/G-like family of proteins (Pfam family PF13400) and a single transmembrane domain as is the case for the *A*. *actinomycetemcomitans* TadG protein. They might have a role in anchoring the pilus to the cell, as suggested for *A*. *actinomycetemcomitans* TadG (Wang and Chen, 2005). In summary, the genetic data support the view that a complex competence pilus or pseudopilus is formed in *M. luteus*, for whose function in transformation the products of both *tad* gene clusters are needed.

By replacing the putative major Flp pilin with a Strep-tagged variant followed by Western blot and immunofluorescence microscopy we could show that, under competence-inducing conditions, Flp-Strep pilin is expressed, processed and assembled into filamentous structures which extend from the surface of *M. luteus* cells. This observation confirmed the *in silico* prediction that Mlut_18170 codes for a Flp prepilin and showed for the first time the existence of Flp assemblages in this organism, which seemed to be expressed only in a small fraction of the population. Expression of Flp pili only under special conditions and only in certain cells are probably the reasons why pili have not been reported before in *M. luteus*. To our knowledge, this is the first direct evidence for the existence of Flp pili in a Gram-positive bacterium.

The shearing/enrichment and mass spectrometry experiments further showed that both *tad* gene clusters need to be intact not only for transformation to occur, but also for the appearance of Flp complexes on the outside of the cells. It is currently unclear why the products of the *tad-1* cluster are required for the formation of Flp pilin complexes, as the *flp*-containing *tad-2* cluster seems to contain a complete set of pilus biogenesis genes. Possible explanations could be that (i) the Flp-like proteins encoded in the *tad-1* cluster (TadE and TadF) are needed as obligatory minor Flp pilus components, or that (ii) TadE and TadF form an accessory oligomeric structure at the base of the pilus (or spanning the cell wall) which is involved in guiding pilus assembly. In our pili preparations, we could not detect by MS any peptide fragments from either TadE, TadE2 or TadF and each of these putative pilins were required for Flp expression on the cell surface, which favors the second hypothesis. Clearly, the role of the TadE- and TadF-like proteins in Gram-positive pilus biogenesis systems needs further investigation.

In contrast to the Gram-negative competence systems, the structure and function of competence pili in Gram-positive bacteria are less studied. So far, two reports have shown that a large macromolecular complex, containing ComGC subunits linked by disulfide bridges, can be found at the surface of competent *B. subtilis* (Chen et al., 2006; Kaufenstein et al., 2011) and one study has recently shown the existence of a “true” transformation pilus, morphologically similar to T4P of Gram-negative bacteria and containing solely ComGC pilin, in *S. pneumoniae* (Laurenceau et al., 2013).

Concerning the regulation of competence development in *M. luteus*, the data presented here, which are in accordance with previous work by Kloos (1969b), suggest that different conditions are of relevance. A clear dependence on the growth phase is evident, as in mid- to late exponential phase the transformation frequencies were orders of magnitude higher than in stationary phase (Figure 1A). It is however unknown if the cell density acts as a trigger via the accumulation of extracellular pheromones (quorum sensing), as has been reported for *S. pneumoniae, B. subtilis* or *V. cholerae* (Morrison and Lee, 2000; Hamoen et al., 2003; Suckow et al., 2011). Other changes of growth parameters, perhaps commencing during exponential growth, such as nutrient availability, pH change, accumulation of products of metabolism, etc. could play a role as well. Nutrient limitation, brought about by dilution of the growth medium, resulted in increased transformation frequency and thus is an important stimulus for competence development in *M. luteus* (Figure 1B). Starvation also stimulates competence in *B. subtilis* or *H. influenzae* (Macfadyen et al., 1998; Claverys et al., 2006). Notably, the underlying regulation must be completely different from the situation in *B. subtilis* because homologs of neither the master regulator nor of any other components of the ComK regulatory cascade could be identified in *M. luteus*.

**Figure 5:**
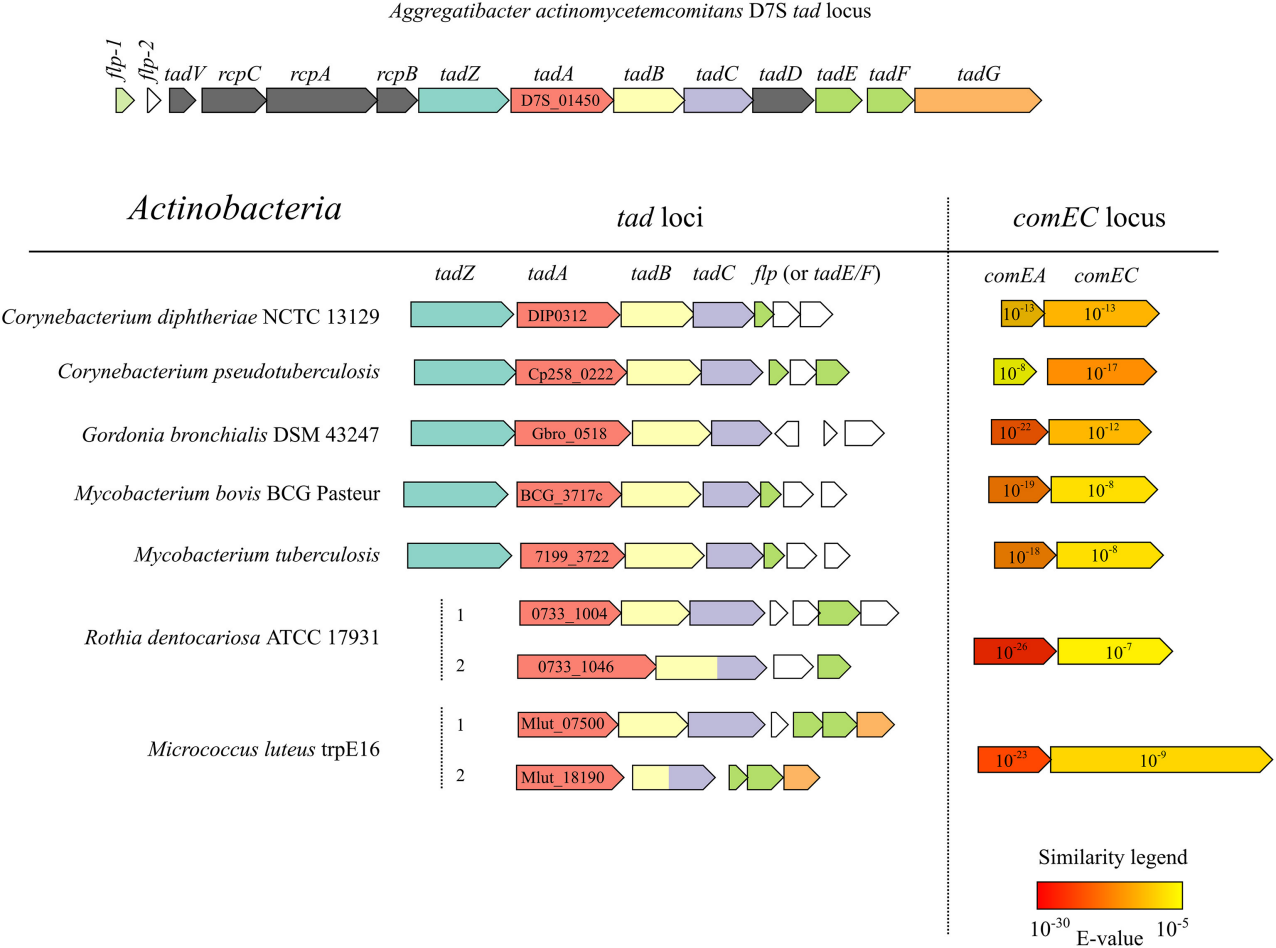
**Physical maps of *tad* and *comEA/EC* loci from the genomes of selected actinobacterial representatives (high-GC Gram-positive) and of the prototypical *tad* locus of *A. actinomycetemcomitans***. ORFs of the same color encode proteins with significant similarity (BLAST *E* < 10^−6^) to the respective *tad* gene products of *A*. *actinomycetemcomitans*. The NCBI locus tags of the predicted TadA homologs are shown and the numbers in the arrows representing ComEA/ComEC homologs are *E*-values of a BLAST search using the full-length *B. subtilis* ComEA/ComEC as queries and all the predicted proteins of the respective organism as a database.

Apart from the two *tad* clusters, the *M. luteus* genome apparently lacks any other genes which may code for T4P or T2SS proteins. Analysis of the distribution of *tad* gene clusters in conjunction with ComEA/ComEC homologs in the available bacterial genomic sequences revealed that this combination occurs frequently in the actinobacterial lineage (Figure 5). Interestingly, *M. luteus* lacks a TadZ ortholog which is in line with the idea that this protein may play a role in polar pilus localization in rod-shaped bacteria [see (Tomich et al., 2007)] while *M. luteus* has a coccoid cell morphology. In contrast to *A. actinomycetemcomitans* and other Gram-negative bacteria, the prepilin peptidase gene (*tadV*) is not closely clustered with the other *tad* genes in the actinobacterial lineage.

Importantly, because both *tad* genes and ComEA/ComEC homologs are widely distributed in the genomes of *Bacteria* and *Archaea* (Claverys and Martin, 2003; Tomich et al., 2007), our finding that Flp pili are a functional part of the transformation apparatus in *M. luteus* extends the set of conserved proteins that can be involved in DNA uptake and may have an impact on the prediction of new transformable species (Johnston et al., 2014), some of them being important human pathogens. We propose *M. luteus* as a well-suited actinobacterial model organism to study Flp pilus/*tad* gene-promoted natural transformation.

## Materials and methods

### Bacterial strains and growth conditions

All strains used in this study are derivatives of *Micrococcus luteus* trpE16, a tryptophan auxotroph of the strain “*Micrococcus lysodeikticus*” ISU (Kloos and Rose, 1970). All *M. luteus* strains (Table 3) were grown in either LB, GMM or CAH media at 30°C in a shaking incubator. Lysogeny broth medium (LB) contained triptone (10 g/L), yeast extract (5 g/L) and NaCl (5 g/L). Glutamate minimal medium (GMM) with glutamate and glucose as the only C-sources was prepared according to Wolin and Naylor (Wolin and Naylor, 1957). For growth of Trp^−^ strains, GMM was supplemented with tryptophan at 0.1 mg/ml. Casein hydrolysate medium (CAH) contained the same components as GMM except glucose, which was replaced with tryptophan-free acid hydrolyzed casein at 5 g/L (EMD Millipore) to allow faster formation of colonies on agar plates. Plates were prepared by supplementing the media with 1.5% (wt/vol) agar.

**Table 3:** ***M. luteus* strains used in this study**.

**Strain**	**Genotype and relevant phenotype**	**Source**
ATCC 27141	Wild type, “*Micrococcus lysodeikticus*” ISU, Trp^+^	Kloos, 1969a
trpE16	*trpE16*, mutagenesis derivative of ATCC 27141, Trp^−^	Kloos and Rose, 1970
ΔcomEC:k	trpE16 Δ12450::*kan*; Kan^R^	Angelov et al., 2013
ΔtadA1:k	trpE16 Δ07500::*kan*; Kan^R^	This study^*^
ΔtadB:k	trpE16 Δ07510::*kan*; Kan^R^	This study^*^
ΔtadC:k	trpE16 Δ07520::*kan*; Kan^R^	This study^*^
Δ753:k	trpE16 Δ07530::*kan*; Kan^R^	This study^*^
Δ754:k	trpE16 Δ07540::*kan*; Kan^R^	This study^*^
Δ755:k	trpE16 Δ07550::*kan*; Kan^R^	This study^*^
Δ756:k	trpE16 Δ07560::*kan*; Kan^R^	This study^*^
Δ757:k	trpE16 Δ07570::*kan*; Kan^R^	This study^*^
Δ758:k	trpE16 Δ07580::*kan*; Kan^R^	This study^*^
Δ1814:k	trpE16 Δ18140::*kan*; Kan^R^	This study^*^
Δ1815:k	trpE16 Δ18150::*kan*; Kan^R^	This study^*^
Δ1816:k	trpE16 Δ18160::*kan*; Kan^R^	This study^*^
Δflp:k	trpE16 Δ18170::*kan*; Kan^R^	This study^*^
ΔtadBC:k	trpE16 Δ18180::*kan*; Kan^R^	This study^*^
ΔtadA2:k	trpE16 Δ18190::*kan*; Kan^R^	This study^*^
ΔtadV:k	trpE16 Δ05160::*kan*; Kan^R^	This study
Flp-Strep:k	trpE16 with *flp* replaced by *flp-strep::kan*; Kan^R^	This study

* *Two versions of these strains were constructed, one with the kanamycin gene in the same orientation as the disrupted ORF and the other with the resistance marker in the opposite orientation*.

### *M. luteus* trpE16 genome sequencing and assembly

Because the trpE16 strain has been obtained by chemical mutagenesis of “*M. lysodeikticus*” ISU in the past, and there have been several changes in the nomenclature of the species, we determined the complete genome sequence of this strain in order to compare it with the type strain for which genome sequence is available, *M. luteus* strain NCTC2665 (Young et al., 2010). Genomic DNA was isolated from the *M. luteus* strain trpE16 (Kloos and Rose, 1970) and used for sequencing on a MiSeq platform (Illumina) with standard manufacturer's protocols (TruSeq LT DNA sample preparation kit, 2 × 150 bp paired-end reads). Mapping assembly was performed with the MIRA assembly program [version 4.0, (35)], using the *M. luteus* NCTC2665 sequence as a reference. The “whole genome shotgun” mapping assembly of *M. luteus* trpE16 was deposited at GenBank under accession number CP007437. Overall, the genome of trpE16 was found to be very similar to that of NCTC2665, displaying only 514 substitutions or small insertions/deletions (0.02% of the genomic sequence). Inspection of the *trpE* genomic regions of both strains revealed that the tryptophan auxotrophy of the trpE16 strain is due to a mutation in the *trpE* gene, a C-T transition leading to a A330V substitution in the anthranilate synthase protein encoded by Mlut_10930.

### Transformation assays

The transformation frequency of *M. luteus* trpE16 and derivative strains was measured by using their tryptophan auxotrophy. For induction of competence, the cultures were grown in GMM until the optical density, measured by absorbance at 600 nm, reached 1.0. The cells (usually 1 ml) were harvested and resuspended in 1 ml of transformation buffer (100 mM CaCl2, 50 mM Tris, pH 7.0). After the addition of transforming DNA (1 μg of a 2528 bp PCR product encompassing the wild type *trpE* gene of *M. luteus* ATCC 27141) and incubation for 30 min at 30°C, 0.1 ml aliquots of appropriate dilutions were plated on CAH- (CAH medium without tryptophan supplementation) for scoring transformants and on CAH+ for determining the total viable cell counts. Control transformations reactions lacking DNA were always performed with the same cell suspensions. Additional control reactions were performed in order to validate our transformation assay. These included transformations with genomic DNA (10 μg) from the trpE16 strain as well such with an analogous PCR product obtained from the trpE16 strain (1 μg of a 2528 bp PCR product encompassing the mutant *trpE* gene of *M. luteus* trpE16). In all cases, the control reactions yielded only spontaneous Trp^+^ revertants with a frequency comparable to the “no DNA” control.

### Construction of gene deletion mutants and reporter strains

For the generation of chromosomal gene disruptions in the parental strain *M. luteus* trpE16, most of the respective coding sequence was exchanged with a kanamycin resistance cassette by homologous recombination. The exchange allele, consisting of approximately 1 kbp upstream and downstream sequences flanking the Tn5 kanamycin resistance gene (Beck et al., 1982), was constructed by assembling *in vitro* equimolar amounts of the three PCR-amplified fragments in a Gibson assembly reaction (Gibson et al., 2009). The Gibson assembly reaction (40 μl) was added to competent *M. luteus* cells in order to introduce the exchange allele by natural transformation and recombinants were selected on LB plates supplemented with kanamycin at 60 μg/ml. The genotype of all constructed strains was verified by PCR and sequencing of the exchanged allele. The genotype of selected strains was additionally confirmed by Southern blot using a biotin-labeled probe complementary to the kanamycin cassette sequence.

The strain carrying the tagged version [*Strep*-tagII, (Schmidt and Skerra, 2007)] of the putative Flp pilus subunit was constructed in a similar way, utilizing a four-fragment Gibson assembly reaction.

### Preparation of enriched *M. luteus* pili and mass spectrometry analysis

For the preparation of enriched high molecular weight protein complexes from extracellular fractions the cells of 1 L cultures grown under competence-inducing conditions were collected by centrifugation (4000 *g*, 20 min), resuspended in 30 ml of PBS buffer and vortexed for 20 min at maximum speed in 50 ml tubes containing 1 cm^3^ of glass beads. After shearing, bacterial cells and debris were pelleted by two rounds of centrifugation (18,000 *g*, 20 min), the supernatant was filtered (0.45 μm) and concentrated to 0.3 ml by ultrafiltration using a Vivaspin 20 unit with a molecular weight cutoff of 100 kDa (Sartorius AG, Germany). The samples were analyzed by Tris-tricine SDS-PAGE carried out with a 16% acrylamide resolving gel with a high degree of crosslinking (*C* = 5%). The acrylamide gels were stained using a silver staining kit for mass spectrometry (Thermo Fisher Scientific, USA). Protein bands of interest were excised, gel slices were reduced and alkylated by 50 mM dithiothreitol and 55 mM chloroacetamide and subjected to in-gel proteolytic digestion using a combination of AspN and GluC (New England Biolabs, USA).

Nanoflow LC-MS/MS was performed by coupling an Eksigent nanoLC-Ultra 1D+ (Eksigent, CA) to an Orbitrap Elite (Thermo Scientific, Germany). Peptides were delivered to a trap column (100 μm × 2 cm, packed in-house with Reprosil-Pur C18-AQ 5 μm resin, Dr. Maisch, Germany) at a flow rate of 5 μL/min in 100% solvent A (0.1% formic acid in HPLC grade water). After 10 min of loading and washing, peptides were transferred to an analytical column (75 μm × 40 cm, packed in-house with Reprosil-Pur C18-GOLD, 3 μm resin, Dr. Maisch, Germany) and separated using a 60 min gradient from 4 to 32% of solvent B (0.1% formic acid, 5% DMSO in acetonitrile; solvent A: 0.1% formic acid, 5% DMSO in water) at 300 nL/min flow rate. The Orbitrap Elite was operated in data dependent mode, automatically switching between MS and MS2. Full scan MS spectra were acquired in the Orbitrap at 30,000 (m/z 400) resolution after accumulation to a target value of 1,000,000. Internal calibration was performed using a DMSO derivate at m/z 401.92272. Tandem mass spectra were generated for up to 10 peptide precursors in the orbitrap for fragmentation using higher energy collision induced dissociation (HCD) at normalized collision energy of 30% and a resolution of 15,000 with a target value of 100,000 charges after accumulation for max 100 ms.

The raw mass spectral data were processed using the MaxQuant software (version 1.4.1.2) for peak detection and quantification (Cox and Mann, 2008). MS/MS spectra were searched against the *Micrococcus luteus* taxonomy restricted Genbank database (version 203, 2236 predicted protein sequences) using the Andromeda search engine (Cox et al., 2011) with the following search parameters: AspN and GluC specificity, up to two missed cleavage sites, carbamidomethylation of cystein residues was set as a fixed modification and N-terminal protein acetylation and methionine oxidation as variable modifications. Mass spectra were recalibrated within MaxQuant (first search 20 ppm precursor tolerance) and subsequently researched with a mass tolerance of 6 ppm, fragment ion mass tolerance was set to 20 ppm. Search results were filtered to a maximum false discovery rate (FDR) of 0.01 for proteins and peptides and a peptide length of at least six amino acids was required.

### Western blotting

Sheared fractions for Western blotting were prepared by collecting the cells of a 20 ml culture by centrifugation for 20 min at 4000 *g* and resuspending them in PBS. The cell suspensions were normalized by absorbance at 600 nm to an optical density of 20, brought to 1 ml and vortexed for 5 min at maximum speed in the presence of glass beads. The glass beads and cells were removed by two rounds of centrifugation (20 min, 13,000 *g*) and samples of the supernatant were run on a Tris-tricine SDS-polyacrylamide gel, transferred to a nitrocellulose membrane and the Flp-Strep protein was detected by Western blotting using alkaline phosphatase-conjugated *Strep*-Tactin (IBA GmbH, Germany) and BCIP-NBT as a chromogenic substrate mix (Thermo Fisher Scientific, USA).

### Fluorescence microscopy

Immunofluorescence microscopy was performed by growing the strain carrying an affinity-tagged version of the Flp prepilin (Flp-Strep:k) under competence-inducing conditions, washing the cells twice in phosphate-buffered saline followed by incubation for 1 h with a 1/250 dilution of Oyster 556-labeled anti-*Strep*-tag antibody (StrepMAB-Classic Oyster 556 conjugate, IBA GmbH, Germany). Microscopy was performed with a Zeiss Axio Imager.M1 epifluorescence microscope equipped with a EC Plan NEOFLUAR oil objective and a HBO100 lamp using a Zeiss filter set 43 (excitation 545/25, emission 605/70) for the visualization of Oyster 556 fluorescence.

### Conflict of interest statement

The authors declare that the research was conducted in the absence of any commercial or financial relationships that could be construed as a potential conflict of interest.
